# NADPH Oxidase 4: A Potential Therapeutic Target of Malignancy

**DOI:** 10.3389/fcell.2022.884412

**Published:** 2022-05-11

**Authors:** Shulei Gong, Shiyang Wang, Mingrui Shao

**Affiliations:** ^1^ Department of Thoracic Surgery, First Affiliated Hospital of China Medical University, Shenyang, China; ^2^ Department of Geriatric Surgery, First Affiliated Hospital of China Medical University, Shenyang, China

**Keywords:** reactive oxygen species, tumor microenevironment, NOX4, cancer, metabolism

## Abstract

Reactive oxygen species (ROS) play a crucial role in the regulation of tumor occurrence and development. As a main source of ROS, NADPH oxidases are key enzymes that mediate electron transport within intracellular membranes. Of the NOX members that have been reported to be dysregulated in a wide variety of tumors, NOX4 is the member to be most frequently expressed. Numerous studies have elucidated that NOX4 gets involved in the regulation of tumor proliferation, metastasis, therapy resistance, tumor-stromal interaction and dysregulated tumor metabolism. In this review, we primarily discussed the biological function of NOX4 in tumorigenesis and progression of multiple cancer models, including its role in activating oncogenic signaling pathways, rewiring the metabolic phenotype and mediating immune response. Besides, the development of NOX4 inhibitors has also been unraveled. Herein, we discussed the interplay between NOX4 and tumorigenesis, proposing NOX4 as a promising therapeutic target waiting for further exploration.

## Introduction

The NOX family of NADPH oxidases are enzymes that mediate electron transport via intracellular membranes (Brandes et al., 2014a). NOX family was initially discovered in the membrane of phagocytes (Bedard et al., 2007). Currently, six members of the NOX family has been identified, including NOX1, NOX3, NOX4, NOX5, DUOX1, and DUOX2 (Vermot et al., 2021). These enzymes share common structure of six transmembrane domains and a C-terminus with a NADPH binding region, with each member exhibiting a specific tissue distribution (Bedard et al., 2007). The regulatory mechanisms for activation of each NOX member are diverse. Various mechanisms have been reported to regulate NOXs activity including post-translational modifications, lipids, calcium level, etc (Rossary et al., 2007; Brandes et al., 2014b; Douda et al., 2015). NOXs also function as reactive oxygen species- (ROS-) producing enzymes to regulate a series of biological function, comprising redox-dependent signaling pathways, oxygen sensor, metabolic reprograming, and immune defense (Bedard and Krause, 2007; Leto et al., 2009). Moreover, downstream ROS production are key regulators of cell differentiation, transformation, growth and death, which are actively engaged in the occurrence and development of multiple cancers (Klaunig, 2018).

Several NOX members have been found to be dysregulated in diverse cancer models, with NOX4 being the member most frequently expressed. Numerous studies have shown that NOX4 plays a crucial role in tumorigenesis and tumor development by supporting cancer cell transformation, proliferation, migration, invasion, and epithelial–mesenchymal transition (EMT). To date, NOX4 has been observed to participate in multiple malignancies, including lung cancer, renal cell cancer (RCC), colorectal cancer (CRC), gastric cancer (GC), pancreatic cancer, glioblastoma, and ovarian cancer, etc (Zeng et al., 2016; Meitzler et al., 2017; Shanmugasundaram et al., 2017; Du et al., 2018; Liu et al., 2021; Shen et al., 2020).

## NOX4, ROS, and Cancer

The sources of ROS are the electron transport chain, producing ROS as a byproduct, as well as NOXs. Besides, endoplasmic reticulum membranes and peroxisomes express enzymes that can generate H_2_O_2_ are also considered as an indispensable supply of intracellular ROS (Brieger et al., 2012). In multiple cancer models, elevated ROS generation has been detected in tumor cells resulting from hypoxic environment, increased metabolic rates or altered redox-related gene expression (Zhang et al., 2016; Wu et al., 2021). Moreover, elevated ROS production also play diverse roles in tumor development, which can activate oncogenic signaling pathway, drive DNA damage and genetic instability, reprogram metabolic phenotype and mediate immune response (Yang et al., 2013; Schieber and Chandel, 2014; Moloney and Cotter, 2018). ROS could regulate a variety of signaling pathways, including PI3K/AKT, hypoxia-inducible factor-1α (HIF-1α), c-myc, NF-κB, and STAT3, and other molecules, thus linking to growth, metastasis, angiogenesis and chemoresistance of tumor (Zhang et al., 2014; Prasad et al., 2017).

**FIGURE 1:**
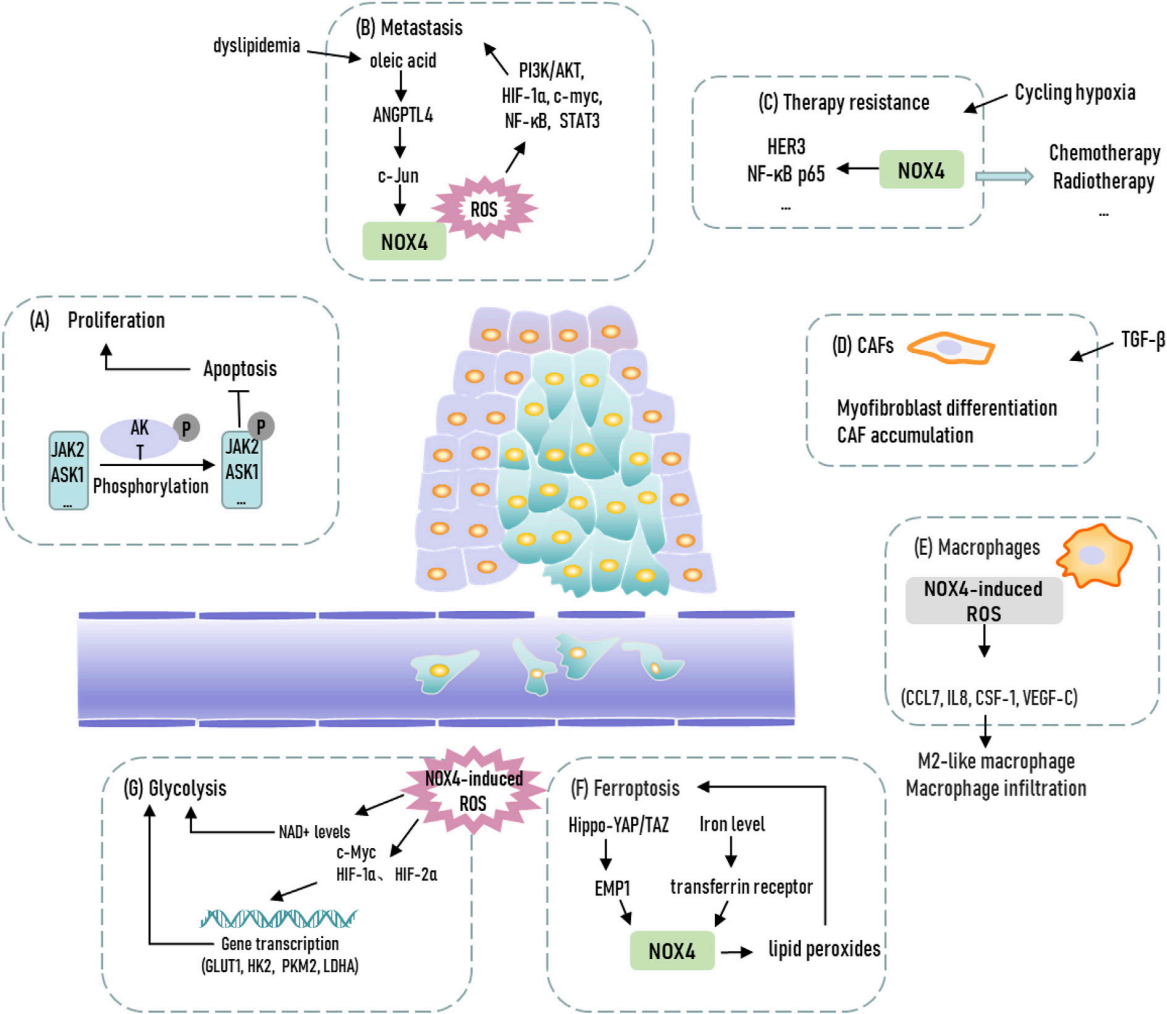
Representation of biological functions of NOX4 in cancer. JAK2, Janus kinase 2; ASK1, apoptosis signal-regulating kinase 1; ANGPTL4, Angiopoetin-like 4; CAFs, cancer-associated fibroblasts; EMP1, epithelial membrane protein 1; GLUT1, glucose transporter 1; HK2, hexokinase 2; PKM2, pyruvate kinase isoform 2; LDHA, lactate dehydrogenase A.

NOX family members are transmembrane proteins that share the capacity to transport electrons across the biological membrane and to produce superoxide and other downstream ROS (Bedard et al., 2007). Conclusively, oxygen is regarded as electron acceptor and superoxide is the product of the electron transfer reaction. Thus, the main biological function of NOX family member is considered as ROS production. NOX4, the most frequently expressed member of NOX family, thus has a potential role in activating diverse signaling pathways and mediating metabolic plasticity through manipulating tumoral ROS level to participate in tumor occurrence and development (Vermot et al., 2021). Therefore, the diverse function of NOX4 and downstream ROS production during tumor progression make it a promising therapeutic target, and it is imperative to thoroughly understand the molecular mechanisms in different cancer models (Figure 1).

## Role of NOX4 in Diverse Malignances

### Lung Cancer

Recent statistics have revealed that lung cancer is the second most frequently diagnosed cancer and the primary cause of cancer-related morality worldwide, comprising both small cell lung cancer and non-small cell lung cancer (NSCLC) (Mao et al., 2016; Sung et al., 2021). Even though significant advances have been achieved in targeted therapy and immunotherapy of NSCLC, which accounts for the majority of lung cancers, exploiting promising therapeutic targets for lung cancer treatment is still urgently needed to improve the prognosis of these patients (Nagano et al., 2019). NOX4 is abundantly expressed in NSCLC tissues and contribute to tumor development through diverse oncogenic mechanisms (Zhang et al., 2019). For instance, NOX4 could reprogram the metabolic phenotype of NSCLC cells to enhance glycolysis and pentose phosphate pathway in A549 and H460 cells. Specifically, NOX4 upregulates the expression of c-Myc, a crucial transcriptional factor for activating glycolytic enzymes, including glucose transporter 1, hexokinase 2, pyruvate kinase isoform 2 and lactate dehydrogenase A, to support the glycolytic phenotype of A549 cells via ROS/PI3K/Akt activation (Zeng et al., 2016; Fang et al., 2019). Nuclear factor erythroid 2-related factor 2 (Nrf2) mediates the modulation of redox adaptation in NOX4-overexpressed NSCLC cells. NOX4-induced ROS maintains the stability of Nrf2 protein by preventing ROS-related proteasomal degradation via PI3K activation (Wu et al., 2017). Besides, NOX4-induced ROS could also elevate cytokine production via PI3K/Akt signaling-dependent manner to increase tumor-associated macrophage infiltration and exert pro-tumor function in NSCLC (Zhang et al., 2019). Cisplatin-based chemotherapy has been regarded as a traditional and primary treatment to prevent the relapse of NSCLC and improve the clinical outcomes of NSCLC patients. However, cisplatin resistance remains a huge obstacle for therapeutic response in NSCLC patients. In NSCLC, EF-hand domain-containing protein D2 (EFHD2) could upregulate NOX4-induced ROS production of A549 and H1299 cells, which ultimately increases transporter ATP-binding cassette subfamily C member 1 expression to elevate cisplatin efflux. A non-steroidal anti-inflammatory drug ibuprofen, which can degrade EFHD2 and thus inhibit EFHD2 expression, could improve the therapeutic response of NSCLC to cisplatin via inhibiting NOX4-ROS-ABCC1 axis (Fan et al., 2020). Hence, NOX4 inhibition may serve as a promising treatment for NSCLC patients.

### Renal Cell Cancer

RCC, which commonly exhibits loss of Von Hippel-Lindau (VHL) suppressor gene, represents the majority of cases with kidney cancer (D'Avella et al., 2020; Sung et al., 2021). Higher NOX4 expression has been found to be correlated with unfavorable survival in RCC (Kaushik et al., 2020). Precious study has revealed that NOX4 expression in RCC tissues mediates the expression and activity of hypoxia-inducible factor 2α (HIF-2α), a crucial transcriptional factor of tumor glycolysis and various malignant behavior, to support renal tumorigenesis (Maranchie and Zhan, 2005; Hoefflin et al., 2020; Singhal et al., 2021). It is worth noting that NOX4 could alter the distribution of HIF-2α via redox adaptation. Specifically, NOX4 silencing in 786-0 and RCC4 NS cells could reduce nuclear accumulation of HIF-2α under both normal and hypoxic oxygen conditions, indicating NOX4 as an alternative activating signal for HIF-2α translocation (Gregg et al., 2014). It is demonstrated that NOX4 also serves as a mitochondrial energetic sensor engaged in reprogramming tumor metabolism for drug resistance. Of note, NOX4 has an ATP-binding motif to directly bind to ATP, which negatively regulates NOX4 activity in VHL-deficient RCC. Further, NOX4-induced ROS in 786-O and A498 cells could reduce the acetylation of pyruvate kinase-M2 isoform from being degraded mediated by P300/CBP-related factor. NOX4 inhibition, through pyruvate kinase-M2, sensitizes 786-O and A498 cells to drug-induced cell death in xenograft models and *ex vivo* cultures (Shanmugasundaram et al., 2017). Recent study also elucidated that NOX4 as a renal-enriched ROS-generating enzyme essential for lipid peroxidation and ferroptosis in RCC. The expression of NOX4 can be induced by activation of Hippo-YAP/TAZ pathway to further enhance lipid peroxidation, which mediates susceptibility of RCC4 and 786O cells to ferroptosis (Yang et al., 2019; Yang and Chi, 2019). Besides, NOX4 could increase hypoxia-induced IL-6 and IL-8 production in RCC, linking NOX4 to inflammation-induced RCC metastasis and making NOX4 a therapeutic target to reduce IL-6- and IL-8-induced inflammation and invasion in RCC (Fitzgerald et al., 2012). According to these observations, NOX4 blockade might aid the development of therapeutic intervention of RCC, especially in TAZ-activated CRC cells.

### Gastrointestinal Cancer

GC is the fifth most frequently diagnosed malignancies and fourth common cause of cancer-related deaths globally (Sung et al., 2021). NOX4 exhibits upregulated expression in GC tumor tissue compared with adjacent normal tissues (Du et al., 2019). Moreover, anoikis-resistant MKN-45 and AGS cells exhibit enhanced malignant phenotypes, which can be attenuated by NOX4 blockade. This can be explained by the finding that detachment from the ECM drives NOX4 overexpression, and NOX4-induced ROS could directly upregulate EGFR expression, further increasing anoikis resistance of GC cells (Du et al., 2018). Besides, NOX4 mediates the upregulation of GLI1, a transcription regulator for the Hedgehog signaling pathway (Doheny et al., 2020). NOX4 overexpression could promote MKN-45 and AGS cell proliferation via activation of the GLI1 pathway, while GLI1 knockdown reverses the malignant phenotype induced by NOX4 overexpression (Tang et al., 2018). Thus, NOX4 is a promising therapeutic target for blockage of GC development.

CRC represents the third most frequent cancer, but second in terms of mortality (Sung et al., 2021). Upregulation of NOX4 expression has been found to be strongly correlated with myofibroblastic cancer-associated fibroblasts (CAFs). In fibroblasts, NOX4 inhibition abrogates ROS production, blocking myofibroblast differentiation and CAF accumulation. NOX4 inhibition can revert the myofibroblastic-CAF phenotype, which indicates NOX4 inhibition as a stromal-targeted approach for CRC (Hanley et al., 2018). Besides, CRC development are highly correlated with metabolic disorders, like dyslipidemia (Chen et al., 2021). It is demonstrated that NOX4 regulate oleic acid (OA)-drived CRC metastasis. OA-induced ANGPTL4 upregulates NOX4 expression via the activation of c-Jun, which further increase ROS production to promote SW480, and HT-29 cell metastasis (Shen et al., 2020). Targeting this ANGPTL4/NOX4 axis may provide promising therapeutic opportunity for dyslipidemia-associated CRC metastasis. Recent study has investigated the role of NOX4 in the survivin-associated adaptive response in colorectal cells. NOX4 inhibition induces the reversal of HCT116 cell TP53 WT and HCT116 cell Mut adaptive responses from pro-survival to radio-sensitization (Murley et al., 2018). Besides, the anti-tumor effect of silver nanoparticles (AgNP) has also been investigated in CRC tumors. AgNPs induce ROS production and endoplasmic reticulum stress responses through NOX4, leading to HCT116 cell apoptosis (Quan et al., 2021). All these evidences prove that NOX4 blockade could be a potential target in CRC treatment.

### Pancreatic Cancer

Pancreatic cancer ranks seventh in terms of mortality globally with a usually poor prognosis, while pancreatic ductal adenocarcinoma (PDAC) accounts for more than 80% of pancreatic cancer cases (Sung et al., 2021). In the membrane of endoplasmic reticulum (ER), high NADPH levels upregulates NOX4 expression. Peroxiredoxin 4 (PRDX4), an antioxidant protein expressed in the ER, is essential for growth and survival of MIAPaCa-2 and PANC-1 cells depending on NOX4-induced ROS production (Jain et al., 2021). In response to hypoxia, HIF-1α could activate NOX4 to elevate the methylation modification of histone H3, thus increasing the transcriptional upregulation of EMT-related marker to enhance metastasis. NOX4 blockade impairs the activation of the HPAC and Panc1 cell survival kinase AKT by attenuating phosphorylation of AKT (Li et al., 2021). NOX4 activates phosphorylation of Janus kinase 2 to exert anti-apoptotic effects. NOX4 activation could impair activity of protein tyrosine phosphatases to increase Janus kinase 2 to impede PaCa cell apoptosis (Lee et al., 2007). NOX4-induced ROS can also protect tumor cells from apoptosis via AKT-dependent phosphorylation of apoptosis signal-regulating kinase 1, thus NOX4 blockade resulting in cell apoptosis (Mochizuki et al., 2006). Oncogenic Kras and inactivation of p16 regulate the expression of NOX4 in PDAC, which could enhance glycolysis by increasing supplement of NAD^+^. NAD^+^ functions as a primary substrate for GAPDH-mediated glycolysis to strengthen PDAC growth (Ju et al., 2017). In cachectic muscles, tumor-induced SIRT1 loss leads to the activation of NF-κB, which upregulates NOX4 expression to exaggerate pancreatic tumor-induced cachexia. Therefore, targeting the Sirt1-NOX4 axis in cachectic muscles may provide a promising opportunity to overcome cachexia in patients with pancreatic cancer.

### Glioblastoma

Glioblastoma is one of the most lethal and common brain tumors, with the 2-years overall survival of glioblastoma patients exhibiting only 25% (Aldape et al., 2003; van Tellingen et al., 2015). Increased NOX4 expression has been observed in glioblastoma tissues (Shono et al., 2008). Cycling hypoxia is an environmental cue for participating in tumor progression. Cycling hypoxic glioblastoma cells exhibit significantly upregulated NOX4 expression and ROS production compared with normoxic cells (Hsieh et al., 2012). Cycling hypoxia drives NOX4 expression to promote resistance to radiotherapy in glioblastoma. Ferroptosis is an important type of programmed cell death and intensively related to iron-associated lipid peroxidation and intracellular homeostasis, which is involved in occurrence and development of tumor. In glioblastoma, increased iron level induced by pseudolaric acid B treatment via upregulation of transferrin receptor could upregulate NOX4 expression, thus leading to accumulated lipid peroxides (Wang et al., 2018). NOX4 also participates in the regulation of TGF-*β*1-drived metabolic rewiring of glioblastoma cells (Su et al., 2021a). NOX4-induced ROS production further increases HIF-1α nuclear accumulation and stability to enhance aerobic glycolysis and EMT program of U87 and A172 cells. NOX4-induced ROS also increases FOXM1 transcription to mediate HIF-1α stabilization to enhance aerobic glycolysis (Su et al., 2021b). These findings have elucidated NOX4 as a crucial factor for supporting malignant behaviors of glioblastoma cells.

### NOX4 and Other Malignancies

NOX4 also participates in the regulation of other cancer types, including prostate cancer, gallbladder cancer, thyroid cancer, and ovarian cancer (Sampson et al., 2018; Helfinger et al., 2019; Pan et al., 2020; Liu et al., 2021). In human prostate cancer, NOX4 mediates TGFβ1-induced activation of primary fibroblasts to acquire a CAF-associated phenotype. NOX4 blockade could diminish CAF-associated marker expression and migrative capabilities of CAFs (Sampson et al., 2018). NOX4 is highly upregulated in gallbladder cancer cells and gallbladder CAFs, which is associated with malignant behaviors and poor prognosis. Specifically, gallbladder CAFs elevate vasculogenic mimicry formation and tumor growth through increasing NOX4 expression via activating IL-6-JAK-STAT3 signaling (Pan et al., 2020). In thyroid cancer, knockdown of NOX4 and p22phox in hypoxia reduces ROS level, thus destabilizing HIF1α to restraining glycolysis and cell growth of TPC-1 cells (Helfinger et al., 2019). NOX4 also mediates the resistance to chemotherapy and radiotherapy in ovarian cancer cells. NOX4 blockade could significantly elevate response of A2780 and OVCAR3 cells to chemotherapy and radiotherapy by downregulating HER3 and NF-κB p65 (Liu et al., 2021). Taken together, NOX4 displays great significance in wide variety of cancers, indicating great potentials of NOX4 inhibitors as anti-tumor therapy.

### Prospects of NOX4 Inhibitors in Cancer Therapy

With the acknowledgements to the role of NOX4 in tumor occurrence and development, NOX4 is an emerging therapeutic target for development of anti-tumor drug. NOX4 inhibitors, including GKT137831, fulvene-5, and diphenyleneiodonium (DPI), are being extensively studied. Currently, GKT137831 is the only NOX4 inhibitors that has been tested in clinical trials. GKT137831 can revert the myofibroblastic-CAF phenotype and promote CD8^+^ T cell infiltration (Hanley et al., 2018). GKT137831 also attenuates prostate cancer cell-driven fibroblast activation to mediate tumor stromal interactions (Sampson et al., 2018). In NSCLC, NOX4 blockade by GKT137831 could reduce pro-tumoral M2-like macrophage and immune infiltration to limit tumor growth (Zhang et al., 2019). In tumors with high CAF levels, GKT137831 can increase immunotherapy response. Moreover, immunotherapy resistance can be impeded by NOX4 blockade and favor prognosis in multiple cancers (Ford et al., 2020). Fulvene-5 has been confirmed to be a potential NOX4 inhibitor. In hemangioma, fulvene-5 impairs NOX4 activity and inhibits hemangioma growth *in vivo* (Bhandarkar et al., 2009). Fulvene-5 could revert adaptive responses of CRC cells from pro-survival to radio-sensitization (Murley et al., 2018). As a pan NOX inhibitor, DPI inhibits phosphorylation of STAT3 and STAT5, and drives PARP1 cleavage in JAK2V617F-positive cells (Lima et al., 2021). DPI can also eliminate ROS production to inhibit tumor progression in ovarian, prostate and lung cancer (Xia et al., 2007; Ma et al., 2021). Even though NOX4 inhibitors have been evaluated in multiple cancer types for its anti-tumor activity *in vitro* and vivo, it is still required to explore deeper to identify the cancer types that can be effectively treated with NOX4 inhibitors. Additionally, clinical trials should be accelerated to evaluate the therapeutic effects of more novel NOX4 inhibitors in different cancer types. Besides, it is necessary to exploit potential biomarkers that sensitize tumor cells for NOX4 blockade. Given the role of NOX4 in ROS production, combination therapies with ROS scavenger should be further explored.

## Conclusion

Precious studies have clearly introduced NOX4 as a key regulator of cancer occurrence and development in multiple cancer models. Multiple underlying mechanisms by which NOX4-induced ROS production have been elucidated. NOX4-induced ROS production could activate various oncogenic signaling pathway, rewire metabolic phenotype of tumors and reprogram the tumor stroma. Tumor microenvironment is a complex and dynamic network, which contains a heterogeneous composition of immune cells, endothelial cells, and fibroblasts, etc. Worth noticing, NOX4 is a key interface to tumor-stromal cell interactions. For instance, NOX4 inhibitor could restrain fibroblast activation resulting from stimulation of surrounding tumor cells, indicating the value of NOX4 blockade as a stromal-targeted strategies. Currently, it is well-recognized that tumor cells co-opt specific immune checkpoints to regulate resistance to immunotherapy (Pardoll, 2012). Specifically, αPD-1/PD-L1 immune-checkpoint immunotherapy have displayed enormous potential in a wide variety of cancer types (Ai et al., 2020). Thus, exploring underlyingly molecular mechanisms of resistance to immunotherapy is critical for developing effective anti-tumor strategies. NOX4 blockade has shown its potential for enhancing the efficacy of immunotherapy, elucidating the therapeutic potential of NOX4 inhibitor in combination with immunotherapy (Ford et al., 2020). Taken together, NOX4, involved in a variety of biological process in cancers, has promising prospective awaiting for further exploration.
